# A randomized controlled trial of curated X exposure for cardiac point of care ultrasound education

**DOI:** 10.1186/s12909-025-07385-3

**Published:** 2025-12-05

**Authors:** Brian Elliott, Sanjay Patel, Laura Elliott, Benjamin Kinnear, Alan Dupre, Hollis Young, John Arthur, Kathryn Burtson

**Affiliations:** 1 Pulmonary and Critical Care Fellow, Department of Pulmonary and Critical Care Medicine, Walter Reed National Military Medical Center, Bethesda, MD 20814 USA; 2 Uniformed Services University of the Health Sciences, Bethesda, MD USA; 3 Boonshoft School of Medicine at Wright State University, Dayton, OH USA; 4 Department of Graduate Medical Education, Riverside Methodist Hospital (OhioHealth), Columbus, OH USA; 5 Clinical Medicine, Ohio University Heritage College of Medicine, Athens, OH USA; 6 PM Pediatric Care, Staff Physician, New Hyde Park, NY USA; 7 Department of Internal Medicine, University of Cincinnati College of Medicine, Cincinnati, OH USA; 8 Keesler Medical Center, Keesler Air Force Base, Biloxi, MS USA; 9 Department of Emergency Medicine, Virginia Commonwealth University, Richmond, VA USA; 10 Department of Internal Medicine, Wright Patterson Medical Center, Wright Patterson Air Force Base, Dayton, OH USA; 11 Department of Internal Medicine, Wright Patterson Medical Center, Wright Patterson Air Force Base, Dayton, OH USA; 12 Department of Internal Medicine, Wright State University Boonshoft School of Medicine, Dayton, OH USA; 13 Department of Internal Medicine, Uniformed Services University, Bethesda, MD USA

**Keywords:** Ultrasound, POCUS, Cardiac, Social media, Medical education, Echocardiography

## Abstract

**Background:**

X (formerly known as Twitter) is a social media platform with a robust online medical education community, including point-of-care ultrasound (POCUS) content.

**Objective:**

Our study aimed to determine if following curated POCUS education accounts on X translated to improved cardiac image interpretation competency.

**Methods:**

We conducted an unblinded parallel-group randomized controlled trial at three internal medicine residency programs and two medical schools. Participants were third- and fourth-year medical students or internal medicine residents. They followed either 10 POCUS education accounts on X (T) or 10 generic medical accounts on X (C) for three months. Participants took a survey and a 15 multiple-choice question assessment before and after the protocol.

**Results:**

Twenty-nine participants completed the protocol, 13 T and 16 C. Mean changes in image interpretation scores were T: -0.02% and C: +0.02%, which did not significantly differ (*p* = 0.46). A simple linear regression evaluating for correlation between average time spent on X and change in image interpretation scores among the T participants did not show a significant correlation (R squared – 0.0002, *p* = 0.96).

**Conclusions:**

Natural X exposure to educational POCUS accounts did not result in statistically improved image interpretation scores, but this outcome was likely affected by limited sample size, different participant experiences, and other variables. Data from this novel trial design can better inform future studies on medical education with social media.

**Supplementary Information:**

The online version contains supplementary material available at 10.1186/s12909-025-07385-3.

## Background

X (formerly known as Twitter) is a social media platform that allows users to share content in 140 characters or less. It has evolved as a valuable educational resource. X uses “push technology,” meaning information, media, and reminders are passively delivered to learners rather than learners actively “pulling” information. X has been shown to improve content retention when used to reinforce curriculum concepts.^1^ Utilization of X has also been shown to improve learner engagement and grades, specifically in undergraduate medical education, with most learners finding it user-friendly and useful.^2^, ^3^.

Beyond the formal extension of curriculum activities, a large informal peer network has developed on X for medical education. This community of users engages in free open-access medical education (FOAMed). FOAMed utilizes one of the central mechanisms of learning theory supplied by social media: connectivism.^4^ There is sparse data investigating if FOAMed translates to improved competency metrics. One pre-post study concluded that X effectively improved medical student electrocardiogram reading skills, but there is a gap in the literature with prospective comparative data.^5^.

Ultrasound image interpretation could be particularly amenable to social media learning, as social media platforms allow easy dissemination of multimedia content like ultrasound video clips and images. This facet likely explains why approximately one-third of emergency ultrasound fellowship programs utilize social media platforms as a pedagogical tool.^6^.

The efficacy of natural social media learning is particularly unclear, as we are not aware of any study that evaluates whether simply following medical education accounts on X leads to measurable improvement in knowledge. Our study aimed to explore this concept, evaluating whether natural exposure to FOAMed on X during undergraduate and graduate medical education leads to improved learner competency in cardiac point-of-care ultrasound (POCUS) image interpretation. We hypothesized that natural exposure to POCUS education accounts on X would result in increased learner competency.

## Methods

### Design and participants

We conducted an unblinded parallel-group randomized controlled trial at three internal medicine residency programs (Wright State University, Walter Reed National Military Medical Center, and Riverside Methodist Hospital/OhioHealth) and two medical schools (Wright State University Boonshoft School of Medicine and Northeast Ohio Medical University) from November 2021 to June 2024. The trial protocol with an a priori analysis plan and timeframe enrollment endpoint of July 2024 was registered on socialscienceregistry.org.^7^.

Eligible participants were 18 or older, had an active status as a third- or fourth-year medical student or an internal medicine resident physician, and could access X. Exclusion criteria included formal training in point-of-care ultrasound outside of usual graduate medical education activities (i.e. certificate or fellowship in point-of-care ultrasound, previous work as an ultrasound technician, etc.), currently following five or more X accounts that consistently posted content related to POCUS, currently following five or more X accounts listed in the intervention arm, or engaging in POCUS education outside of typical medical education curricula during the study period.

### Randomization and enrollment

We recruited participants via email, including a study overview and exclusion criteria. In June 2023, we were awarded grant funding to aid in recruitment for the study, so participants enrolling after June 2023 were offered $40 for their participation.

Investigator LE conducted randomization, pre-assigning 120 enrollments using permuted block randomization in blocks of four via GraphPad QuickCalcs Web site: http://www.graphpad.com/quickcalcs/ (accessed October 2021), stratified by resident or medical student status. For each enrollment, LE was blinded to the participant.

### Trial protocol

The protocol was conducted remotely through the REDCap data management software.^8^, ^8^ After randomization and enrollment, participants completed the short survey and multiple-choice question (MCQ) assessment (Supplement 1 with images from thepocusatlas.com used under a CC BY-NC 4.0 license). Then, they were instructed to either follow 10 X accounts that regularly post POCUS content or 10 X accounts that post general medical content. The list of accounts is available in Supplement 2.

Participants were asked to use X for at least two hours per week during the study without explicit recommendations for engaging with the content or limitations on which additional accounts to follow during the study. The two-hour recommendation was based on an informal focus group of medical students and residents, in which they were asked what a feasible time recommendation would be.

Every three weeks, participants received automated emails with a survey to log their X use during the preceding three weeks. The log included instructions to view and log application use metrics tracked by Android or iPhone software. The MCQ assessment followed the final log at 12 weeks. All emails were programmed to send up to two reminders every two days if the participant did not complete the survey. Participants were included in the final analysis if they completed a pre-and post-study assessment and at least one interim log.

### Assessment creation and evaluation

We chose to create a novel MCQ assessment because we could not find one described that had substantial validity evidence and met our purpose. Authors AD, BE, BK, KB, and LE created the assessment. Their expertise includes certification through the Society of Hospital Medicine POCUS program (BE), a master’s in education (BK), a certificate in health professions education (KB), and research background in POCUS or assessment creation (BE, BK, KB, and LE).

The baseline assessment data was used to evaluate the assessment characteristics. We calculated characteristics using a discrimination index with a standard 27% top and bottom threshold, proportion correct, and point biserial correlations.^9^ We evaluated reliability with a test-retest comparison among control arm participants (baseline assessment vs. final assessment) with Pearson’s correlation coefficient *r*.

### Statistical analysis

We determined the a priori sample size for detecting a difference between the two study arms regarding our primary endpoint: change in MCQ assessment score from pre- to post-protocol. To detect a 20% improvement in the intervention arm, it estimated that a sample size of 32 would be required to detect a difference at the 95% confidence interval with 80% power.

Descriptive statistics were done with Microsoft Excel version 2312. Statistical comparisons were done with GraphPad Prism version 10.1.2 for Windows. Continuous variables were compared using the Mann-Whitney test. Categorical variables were compared using Fisher’s exact test. To evaluate the dose-response relationship, we performed a simple linear regression analysis to evaluate the correlation between the average minutes spent on X per 3 weeks and the change in image interpretation scores pre-post among intervention arm participants.

### Ethics approval and consent to participate

The study was reviewed by the Wright-Patterson Medical Center Institutional Review Board and approved under the protocol # FWP202110021E. Participants signed an informed consent before voluntarily participating in the study, and they were free to withdraw at any time.

## Results

Fifty-four participants enrolled in the study and underwent randomization: 27 were assigned to the interventional arm, and 27 were assigned to the control arm. Of these, 13 completed the interventional arm protocol, and 16 completed the control arm protocol. The rest were lost to follow-up or requested to withdraw from the study. Of the 34 participants enrolled before the $40 incentives, 11 (33%) completed the protocol. Whereas 18 (90%) participants enrolled after the inclusion of the $40 incentive completed the protocol.

The study population’s baseline characteristics, including their baseline image interpretation assessment score, were not significantly different between the intervention and control arms, as shown in Table 1.

**Table 1:** Baseline characteristics. Categorical variables represented as count (percentage), continuous variables represented as median (interquartile range)

Variable	X *n* = 13	Control *n* = 16	*p* value
Medical student	6 (46)	6 (38)	0.72
Female	6 (46)	6 (38)	0.72
Year enrolled			0.16
2021	1 (8)	1 (6)	
2022	2 (15)	7 (44)	
2023	10 (77)	7 (44)	
2024	0 (0)	1 (6)	
Accounts following	123 (33–257)	52 (2-175)	0.28
Assessment score	40 (33–50)	46 (40–60)	0.16

**Fig. 1:**
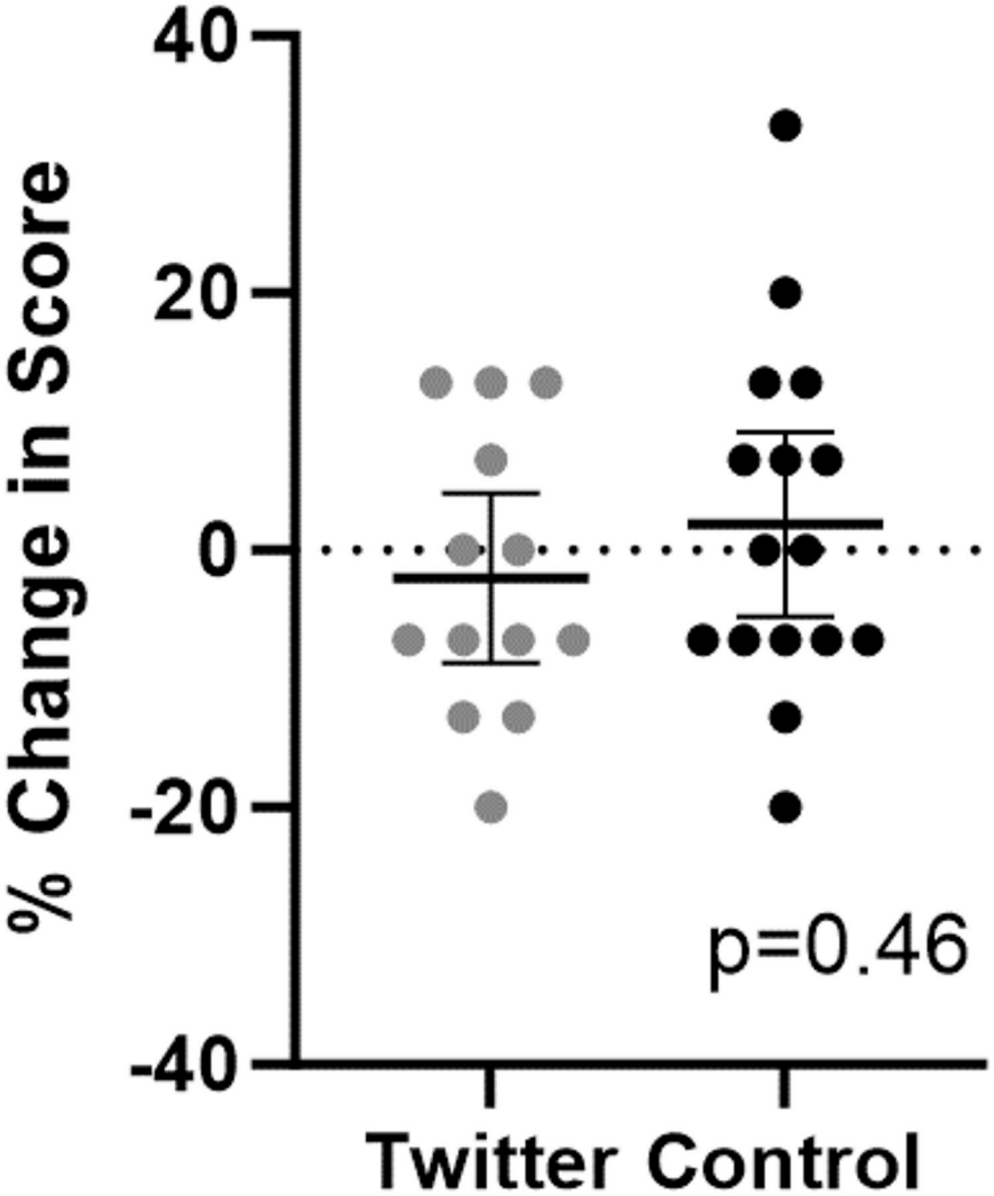
The primary outcome of change in image interpretation scores. The solid bars represent the mean, and the intervals represent the 95% confidence interval. Dots represent individual values

**Fig. 2:**
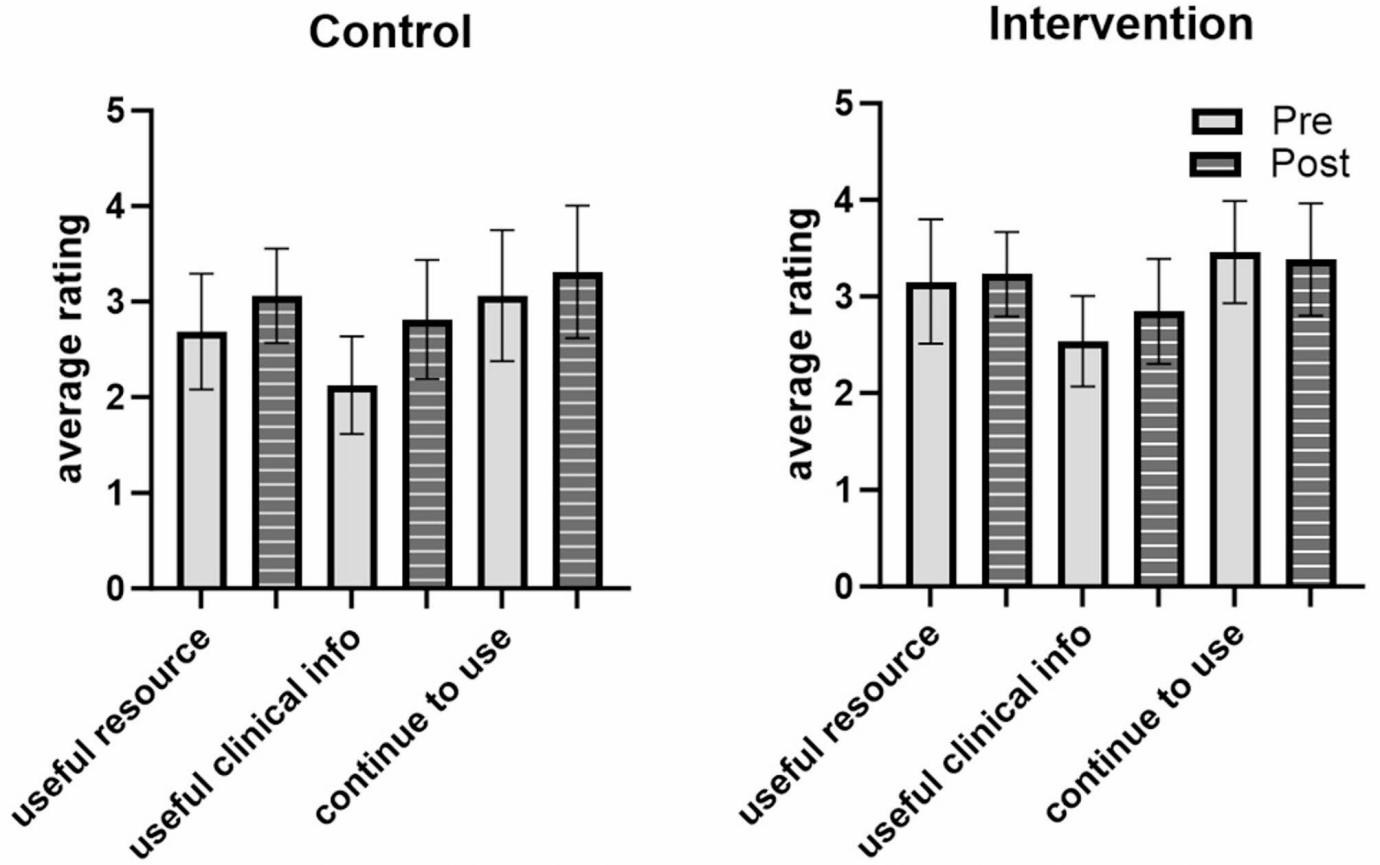
Perception ratings. Participants rated whether they felt X was a useful educational resource, contained useful clinical information, and whether they anticipate continuing to use it for educational purposes. Ratings based on a Likert scale where 5 = strongly agree, 4 = agree, 3 = neutral, 2 = disagree, 1 = strongly disagree. Bars represent the mean and intervals represent the 95% confidence interval of the mean

The two groups had no significant difference in the primary outcome change in image interpretation scores (Fig. 1). Participant perceptions did not significantly differ between study arms and within study arms pre- to post-protocol (Fig. 2).

Interim log results are shown in Table 2. Notably, the average weekly time on X did not significantly differ between the groups. Participants averaged approximately one hour of weekly X use during the study period, below our recommended usage time of two hours.

**Table 2:** Interim log results. Categorical variables represented as count (percentage), continuous variables represented as median (interquartile range). Participants were given interim logs every 3 weeks and asked to log their total minutes on X, average use time represents the average of this variable. “New follows to accounts that post cardiac POCUS content” represents the number of accounts that participants voluntarily followed during the study period that post cardiac POCUS content, in addition to those provided at enrollment. “Engaged with content” represents the number of participants who indicated they commented on posts

Variable	X *n* = 13		Control *n* = 16	*p* value
Count of logs completed				
1	0 (0%)	0 (0%)		
2	0 (0%)	2 (13%)		
3	2 (15%)	1 (6%)		
4	11 (85%)	13 (81%)		
Median use time (min) over 3 weeks	115 (36–367)	210 (63–322)		0.98
Engaged with content	5 (38%)	1 (6%)		0.06
New follows to accounts that post cardiac POCUS content	2 (0–8)	0 (0–1)		0.18
Median time (min) receiving ancillary POCUS education	18 (1–26)	9 (0–29)		0.89

The simple linear regression evaluating the correlation between average time spent on X and change in image interpretation scores did not show a significant correlation (R squared—0.0002, *p* = 0.96).

Participants also indicated which aspects of X were educational. Across both arms, 16 (55%) felt it helped stay current with new medical literature, 13 (45%) indicated the multiple-choice questions posted by X accounts were educationally beneficial, 22 (76%) indicated the threads posted that explain concepts were educationally beneficial, 23 (79%) indicated the clinical images and photos posted were educationally beneficial, and 14 (48%) indicated the networking on X was useful.

### Assessment characteristics

Table 3 reports the assessment characteristics. Notably, no items had a negative discrimination index, and 80% of test items had point biserial correlations greater than 0.20 (the recommended cutoff for good items).^9^ The Pearson *r* for the test-retest reliability comparison showed a high-grade statistically significant correlation of *r* = 0.72; *p* = 0.002.

**Table 3:** Psychometric results from assessment items

Question	Proportion correct	Discrimination index	Point biserial correlation
1	0.66	0.38	0.24
2	0.52	0.38	0.45
3	0.07	0.25	0.23
4	0.79	0.25	0.24
5	0.21	0.00	0.17
6	0.28	0.00	0.15
7	0.62	0.38	0.33
8	0.76	0.50	0.44
9	0.21	0.38	0.32
10	0.79	0.50	0.50
11	0.24	0.25	0.16
12	0.66	0.63	0.53
13	0.28	0.38	0.42
14	0.31	0.63	0.59
15	0.31	0.63	0.56
Average	0.45	0.37	0.35

## Discussion

In this multicenter randomized controlled trial, we investigated the impact of X use on cardiac POCUS image interpretation scores among medical students and internal medicine residents. Contrary to previous studies that reported improvements in medical education outcomes associated with X use, our results showed no significant benefit in image interpretation scores for participants who followed POCUS accounts.^2^, ^5^ A key distinction between our study and previous positive findings is that we examined the effects of curated X exposure, where participants’ activity mimicked real-world scenarios in which medical students and residents follow educational accounts independently, outside of formal curricula. Interestingly, our participant questionnaire data revealed that participants perceived X use as somewhat educational, which is consistent with a large survey that showed health sciences students hold positive views about the educational value of social networking sites.^10^ However, this perceived educational benefit did not translate to significant improvements in image interpretation scores, suggesting a potential disconnect between perceived learning benefits and actual competency gains. Further research is needed to determine whether this disparity represents a genuine mismatch between perception and measurable competency or a type II error due to limitations in our study, including difficulties in tracking covariates, high attrition rates, and a small sample size. These limitations restrict our ability to draw definitive conclusions about the effectiveness of X use for POCUS education, highlighting the need for more rigorous and well-powered studies to explore this question.

The widespread use of social media for medical education necessitates well-designed studies evaluating its efficacy, and the encountered pragmatic issues in this novel study design can better inform future prospective studies on social media education. The first pragmatic issue encountered was tracking interventional covariates. Our participants used X less than recommended (an average of one hour weekly versus the recommendation of two hours weekly), roughly equivalent to 12 h throughout the protocol. Previous literature has shown that cardiac image interpretation can be improved within this timeframe. Hospitalists were able to improve their image interpretation with 6 h of echocardiogram teaching.^11^ An 8-hour curriculum for preclinical medical students was similarly able to improve cardiac POCUS competency.^12^ Curricula for internal medicine residents have shown that averaging just 90–120 min of education is associated with improved competency scores.^13^, ^14^ Since educational posts on X are interspersed with non-educational posts and represent a method of “micro-learning,” or learning online material in short spurts of 15 min or less, the total learning time in our protocol was likely significantly less than the total X time of 12 h.^15^, ^16^ In addition, posts on X are delivered to users by algorithms based on post engagement and view time, creating several other variables that affect the true POCUS exposure for each participant. Thus, the total POCUS learning time could not effectively be tracked and could have been insufficient to gain competency. To help mitigate this absent covariate, we performed a dose-response linear regression that did not find that more time on X was associated with more improvement. We also tracked the fraction of user’s followed accounts that were POCUS accounts. Still, these can be faulty indirect measures of educational exposure. The easiest fix to this issue in future studies would be studying social media in an alternative, controlled platform. For example, using WhatsApp or similar messaging platforms in which an instructor could entirely control the exposure of participants. In X and similar platforms, the exact educational exposure of regular users is uncontrolled, diluted, fluctuating, and cannot be directly tracked. One potential strategy to mitigate this issue would be to enroll participants with new accounts that only follow the accounts studied in the protocol. Other mitigation strategies could include moderated discussions, instructor-led engagement, or scheduled content posting. These would not entirely remove outside content but would better control direct educational exposure and could allow for more meaningful per-protocol and dose-response analyses. The reason we did not choose these methodologies is that our research question was to explore whether the natural use of X translated to meaningful competency improvements, so our protocol was designed with existing educational accounts, and participants were instructed to use X as they see fit without content control. Our experience and attrition rate suggest that using this strategy and attempting to measure all covariates by increasing questionnaire data seems infeasible.

Slow enrollment and attrition rates were another pragmatic issue encountered. Some of the recruitable population that was already using X were ineligible as they were already following educational POCUS accounts. Once enrolled, we found that without financial incentives 70% of the sample withdrew or stopped responding to questionnaires throughout the 3-month study period. We added $40 financial incentives which dramatically improved this issue but was still insufficient to reach our a priori sample size prior to ending the trial. The incentive introduces potential bias in our sample, the attrition rates limit the effects of randomization, and not reaching predetermined sample size estimates limits definitive conclusions. Based on this experience, future studies should employ financial incentives of similar magnitude, if not more, to reach predetermined goals.

Our experience with the trial highlighted the dynamic and unpredictable nature of FOAMed. During the study, some accounts ceased posting, while others varied in their posting frequency, leading to inconsistent exposure to educational content among participants. Furthermore, the X platform underwent a significant change in ownership in October 2022, which has had a profound impact on its academic community. A recent survey of over 9,000 scientists found that 54% had decreased their use of X in 2023, suggesting a potential decline in engagement and content sharing.^17^ Although there is limited data on the specific effects of this change on medical education accounts, it is likely that similar reductions in posts and interactions have occurred within this community. These fluctuations in the platform’s activity and content contributed to substantial variability in the participant experience, which may have influenced our outcomes. To minimize this variability, future studies could consider enrolling participants simultaneously to ensure a more uniform experience. However, the constantly evolving nature of social media poses a challenge to achieving external validity.

In addition to these limitations, our study had several other methodological constraints. Notably, the assessment tool used in our study lacked established validity evidence, which limits the reliability and generalizability of our findings. Although we evaluated the assessment’s internal metrics, its lack of external validation undermines the confidence in its results. Moreover, the assessment required some clinical integration, which may have introduced extraneous variables and reduced its specificity to pure image interpretation. Another potential limitation was the overlap between the control and treatment groups, as control group accounts may have posted educational POCUS content that overlapped with the intervention. Finally, our participants had some prior knowledge of POCUS, as evidenced by their baseline assessment results, which may have influenced the outcomes. It is possible that the results would differ in a population with less prior training or experience.

In light of the study’s limitations, the null findings of this randomized controlled trial investigating the use of X for informal education must be interpreted with caution. While theoretical frameworks discuss how social media may have educational benefits, empirical evidence demonstrating a clear link between social media use and measurable improvements in learner competency is scarce. Our study’s null findings could suggest that informal learning on social media may be insufficient to drive significant gains in competency, but the methodological challenges we encountered limit the conclusiveness of our results. Nevertheless, our experience highlights the importance of addressing pragmatic issues in the design and implementation of future trials examining the efficacy of informal social media education. By acknowledging and addressing these challenges, our study provides valuable insights that can inform the development of larger, more robust trials to investigate the potential of social media as a medical education tool.

## Conclusions

Although curated exposure to cardiac POCUS educational content on X did not result in measurable improvements in image interpretation competency, this study serves as a pilot investigation into the feasibility and limitations of studying social media platforms for medical education with randomized trials. Our findings provide important groundwork for designing future trials with more controlled exposure and enhanced tracking of educational engagement. Importantly, this study highlights the challenges of conducting randomized trials involving informal social media use, where educational exposure is algorithm-driven, variable, and difficult to quantify. Future studies may benefit from alternative platforms with greater content control or hybrid models combining informal and formal learning strategies.

## Electronic supplementary material

Below is the link to the electronic supplementary material.

Supplementary Material 1

Supplementary Material 2

## Data Availability

Raw data generated from this study is available by reasonable request. Please contact brian.elliott@wright.edu to submit a request.

## Declarations

### Author Verification

All authors had access to the data and a role in writing in the manuscript.

## References

[1] Blessing SB, Blessing JS, Fleck BKB. Using Twitter to reinforce classroom concepts. Teach Psychol. 2012;39(4):268–71. 10.1177/0098628312461484

[2] Diug B, Kendal E, Ilic D. Evaluating the use of Twitter as a tool to increase engagement in medical education. Educ Health. 2016;29(3):223–30. 10.4103/1357-6283.204216 28406107

[3] Bahner DP, Adkins E, Patel N, Donley C, Nagel R, Kman NE. How we use social media to supplement a novel curriculum in medical education. Med Teach. 2012;34(6):439–44. 22449268 10.3109/0142159X.2012.668245

[4] Flynn L, Jalali A, Moreau KA. Learning theory and its application to the use of social media in medical education. Postgrad Med J. 2015;91:556–60. 26275427 10.1136/postgradmedj-2015-133358

[5] López-Prado A, Miramontes-González P, Martín-Escudero JC, Pérez-Castrillón JL, Dueñas-Laita A, Rollán MJ, Corral-Gudino L. Effectiveness of Twitter threads to improve medical student electrocardiogram (ECG) Reading-Skills. The TwittUVa-ECG Non-Randomized Pre-Post study. Med Sci Educ. 2023;33(6):1359–69. 38188417 10.1007/s40670-023-01885-x PMC10767012

[6] Amini R, Wang JB, Trueger NS, Hoyer R, Adhikari S. Use of social media in emergency ultrasound fellowship programs. AEM Educ Train. 2017;1(1):27–33. 10.1002/aet2.10005. Published 2017 Jan 19. 30051005 10.1002/aet2.10005 PMC6001491

[7] Elliott B. A randomized controlled trial of self-directed learning with Twitter for ultrasound image interpretation. AEA RCT Registry. 2023.

[8] Harris PA, Taylor R, Thielke R, Payne J, Gonzalez N, Conde JG. Research electronic data capture (REDCap)—a metadata-driven methodology and workflow process for providing translational research informatics support. J Biomed Inform. 2009;42(2):377–81. 18929686 10.1016/j.jbi.2008.08.010 PMC2700030

[9] Yudkowsky R, Park YS, Downing SM, editors. Assessment in health professions education. New York, NY: Routledge; 2019;26.

[10] Dash NR, Hasswan AA, Dias JM, Abdullah N, Eladl MA, Khalaf K, Farooq A, Guraya SY. The educational use of social networking sites among medical and health sciences students: a cross campus interventional study. BMC Med Educ. 2022;22(1):525. 35786406 10.1186/s12909-022-03569-3 PMC9251038

[11] Martin LD, Howell EE, Ziegelstein RC, Martire C, Whiting-O’Keefe QE, Shapiro EP, Hellmann DB. Hand-carried ultrasound performed by hospitalists: does it improve the cardiac physical examination? Am J Med. 2009;122(1):35–41. 19114170 10.1016/j.amjmed.2008.07.022

[12] Kumar A, Barman N, Lurie J, He H, Goldman M, McCullough SA. Development of a point-of-care cardiovascular ultrasound program for preclinical medical students. J Am Soc Echocardiogr. 2018;31(9):1064–6. 30180938 10.1016/j.echo.2018.05.008

[13] Gopal D, Baston C, Adusumalli S, Jagasia D, Prenner S. Focused cardiac ultrasound curriculum for internal medicine residents. POCUS J. 2021;6(1):29–32. 36895500 10.24908/pocus.v6i1.14759 PMC9979930

[14] Mellor TE, Junga Z, Ordway S, Hunter T, Shimeall WT, Krajnik S, Tibbs L, Mikita J, Zeman J, Clark P. Not just hocus POCUS: implementation of a point of care ultrasound curriculum for internal medicine trainees at a large residency program. Mil Med. 2019;184(11–12):901–6. 31125075 10.1093/milmed/usz124

[15] Palmon I, Brown CS, Highet A, Kulick AA, Barrett ME, Cassidy DE, Herman AE, Gomez-Rexrode AE, O’Reggio R, Sonnenday C, Waits SA. Microlearning and social media: a novel approach to video-based learning and surgical education. J Graduate Med Educ. 2021;13(3):323–6. 10.4300/JGME-D-20-01562.1 PMC8207915 34178254

[16] Wakam GK, Palmon I, Kulick AA, Lark M, Sonnenday CJ, Waits SA. Adapting to the times: combining microlearning videos and Twitter to teach surgical technique. J Surg Educ. 2022;79(4):850–4. 35227624 10.1016/j.jsurg.2022.02.001

[17] Valero MV. Thousands of scientists are cutting back on Twitter. Nature. 2023;620:482.

