# Supplementary material for: A randomized controlled trial of curated X exposure for cardiac point of care ultrasound education
Source: BMC Med Educ. 2025 Dec 5;26:42. doi: 10.1186/s12909-025-07385-3 (PMC12797907; doi:10.1186/s12909-025-07385-3)
Supplement: Supplementary file 1 — Supplementary Material 1 [file 12909_2025_7385_MOESM1_ESM.pdf]

Survey and Assessment

Please complete the survey below.

Thank you!

SURVEY

How many accounts do you currently follow on Twitter?

____

How do you view Twitter as a medical education resource?

- ◯ Almost no educational value
- ◯ A little bit of educational value
- ◯ Some educational value
- ◯ Quite a bit of educational value
- ◯ Great educational value

How often do you learn concepts from Twitter that are applicable to clinical practice?

- ◯ Almost never
- ◯ Once in a while
- ◯ Sometimes
- ◯ Often
- ◯ Almost always

How likely are you to use Twitter as an educational resource?

- ◯ Very unlikely
- ◯ Somewhat unlikely
- ◯ Unsure
- ◯ Somewhat likely
- ◯ Very likely

What factors contribute to your decision not to continue to use Twitter as an educational resource?

- ☐ Non-medical content is too distracting
- ☐ Medical content was not very educational
- ☐ I do not like the platform
- ☐ I already have a surplus of educational resources
- ☐ other (describe in text box below)

____

What do you feel are the educational aspects of Twitter?

- ☐ Staying up-to-date on current research
- ☐ Multiple choice clinical questions posted on Twitter
- ☐ Twitter threads explaining concepts
- ☐ Images/videos posted on Twitter
- ☐ Networking with other healthcare professionals
- ☐ Other
- ☐ None

____

ASSESSMENT

Please use the following image to answer question number 1.

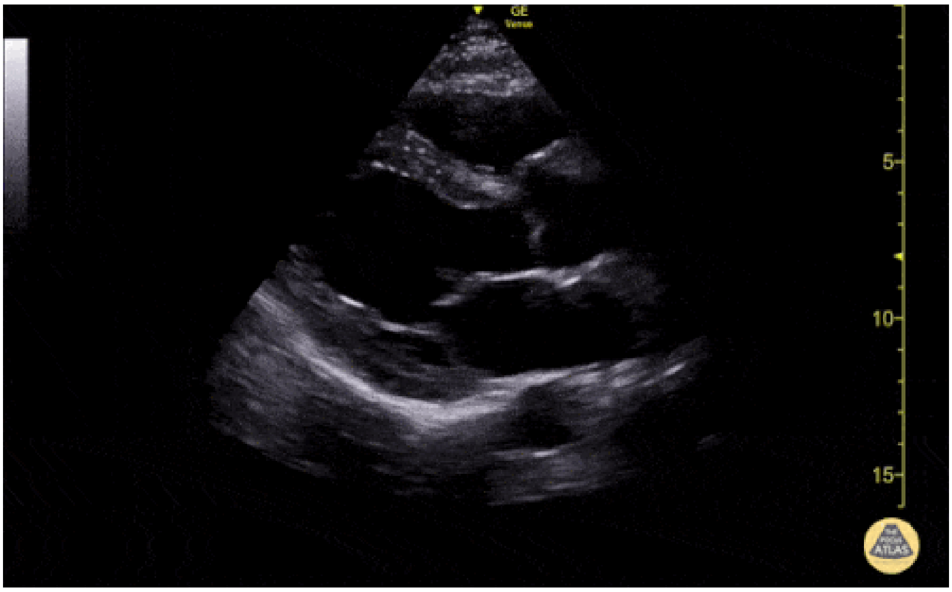

1. Which can be confirmed based on the above image?

- ◯ Normal left ventricular systolic function
- ◯ Dilated left ventricle
- ◯ Reduced left ventricular systolic function
- ◯ Bicuspid aortic valve

Please use the following image to answer question number 2.

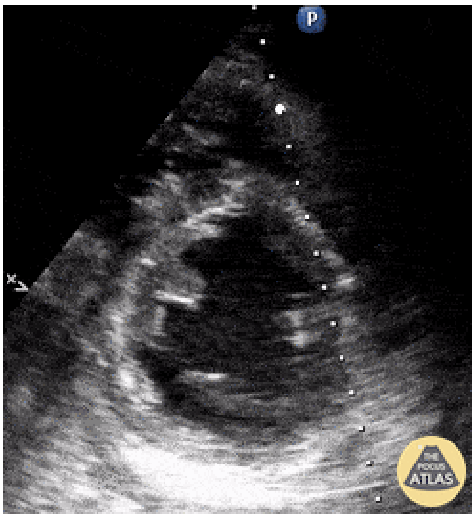

2. What is present?

- ◯ Normal left ventricular systolic function
- ◯ Markedly reduced left ventricular systolic function
- ◯ Pericardial effusion
- ◯ Septal bounce

Please use the following image to answer question number 3.

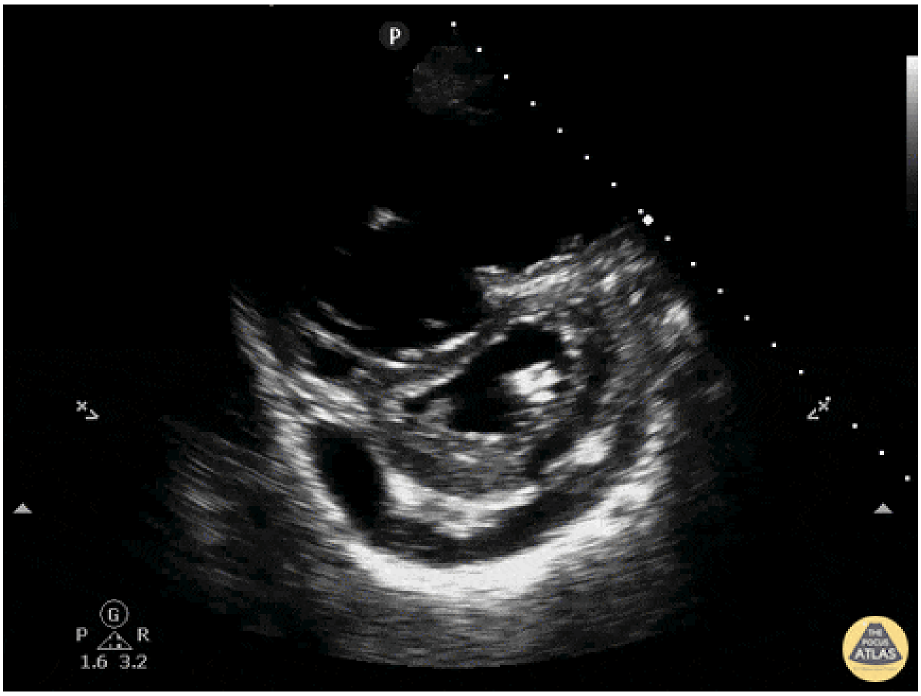

3. Which findings are suggested by the above image.

- ◯ Mitral valve vegetation
- ◯ Pericardial effusion with cardiac tamponade
- ◯ Left ventricular hypertrophy
- ◯ Severely elevated right ventricular pressures

Please use the following image to answer question number 4.

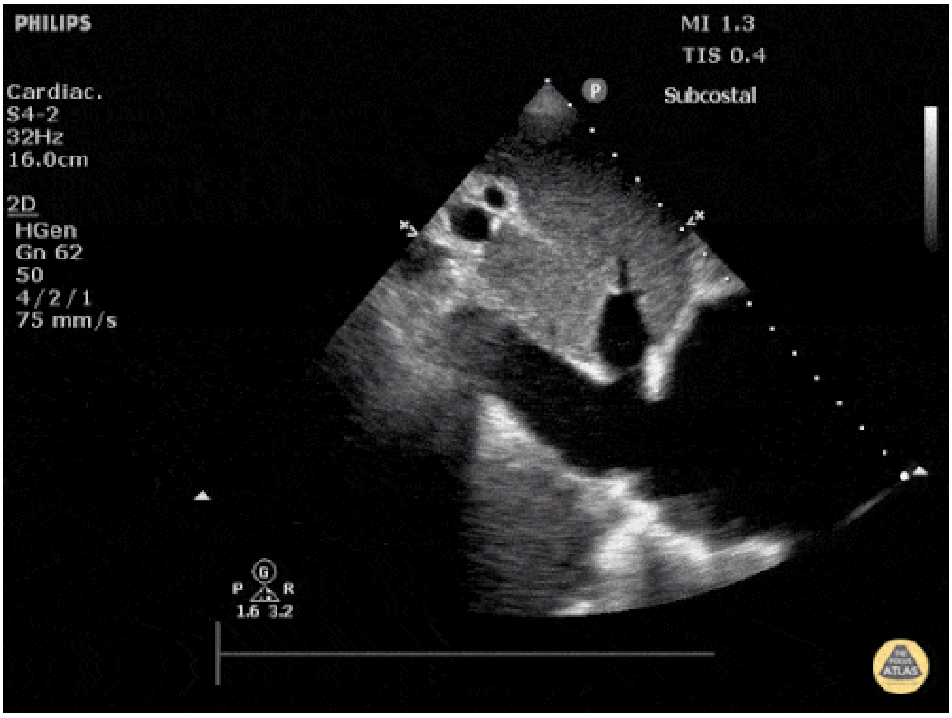

4. Which is suggested by the above image?

- ◯ The patient is euvolemic
- ◯ The patient is hypervolemic
- ◯ The patient is hypovolemic

The following images are obtained from the same patient, please use them to answer question number 5.

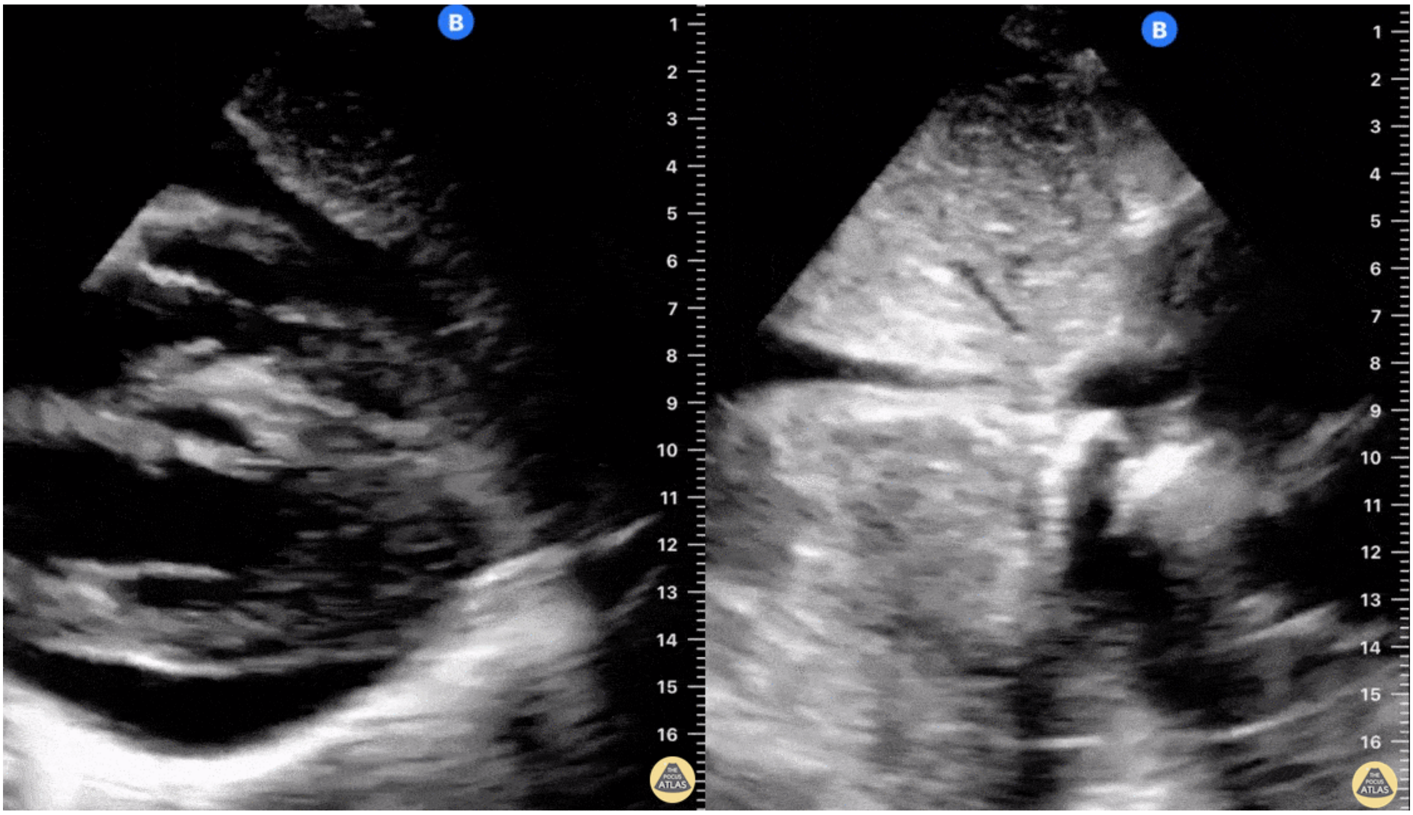

5. Which are suggested by the above images?

- ◯ Pericardial effusion with evidence of tamponade physiology
- ◯ Pericardial effusion without evidence of tamponade physiology
- ◯ Pleural effusion with mass effect
- ◯ Pleural effusion without evidence of mass effect

Please use the following image to answer question number 6.

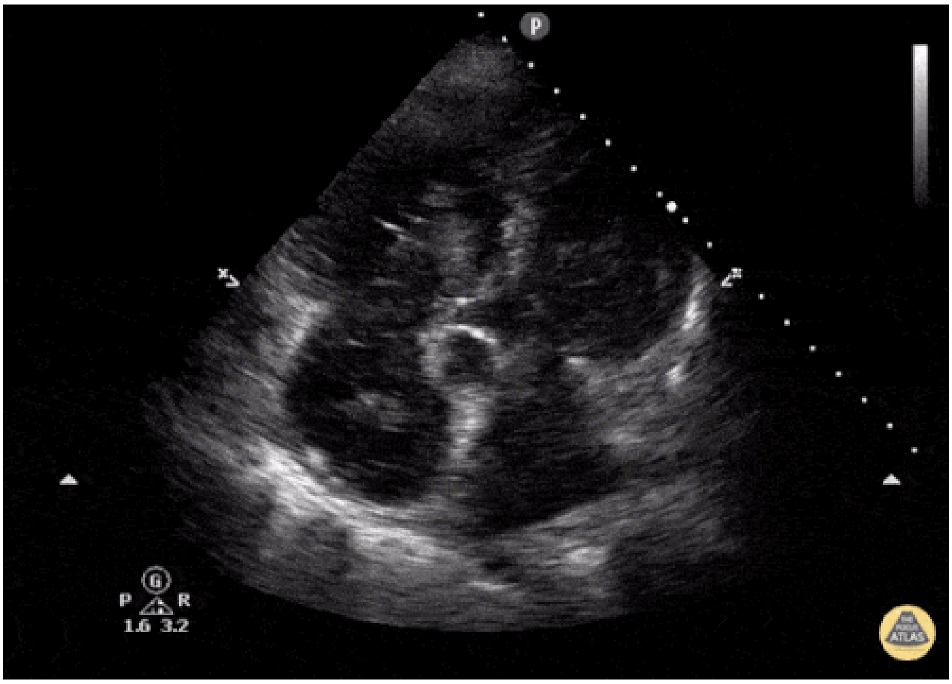

6. Which is present in the above image?

- ◯ Normal findings after injection of agitated saline (bubble study)
- ◯ Abnormal findings after injection of agitated saline (bubble study)
- ◯ Left atrial mass
- ◯ Right atrial mass

Please use the following image to answer question number 7.

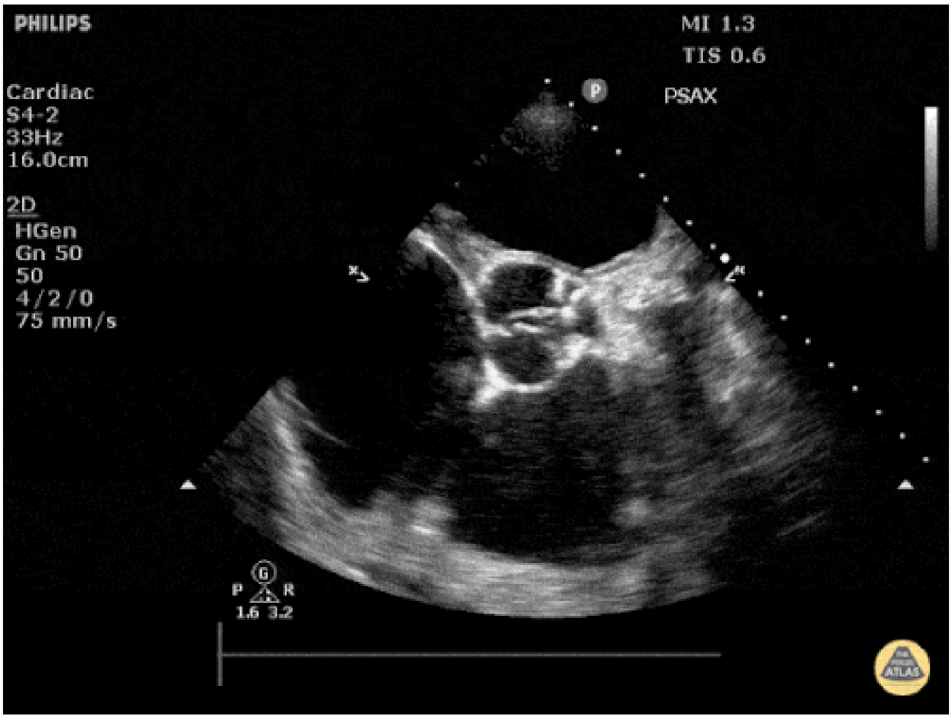

7. Which is suggested by the above image?

- ◯ Left atrial enlargement
- ◯ Aortic valve vegetation
- ◯ Aortic stenosis
- ◯ Right ventricular enlargement

Please use the following image to answer question number 8.

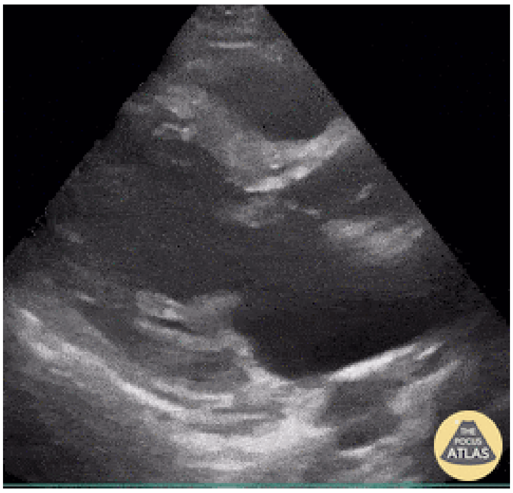

8. Which valve appears abnormal?

- ◯ Tricuspid
- ◯ Aortic
- ◯ Pulmonic
- ◯ Mitral

Please use the following image to answer question number 9.

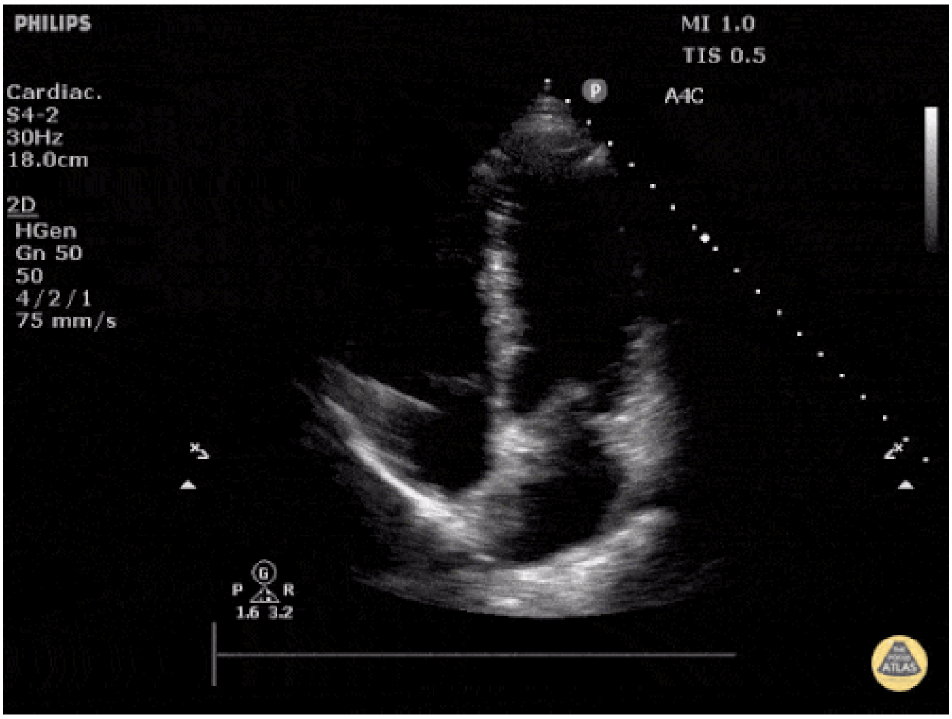

9. Which of the following is demonstrated?

◯ Reduced left ventricular systolic function
◯ Elevated right ventricular pressure
◯ Flail leaflet
◯ Normal study

Please use the following image to answer question number 10.

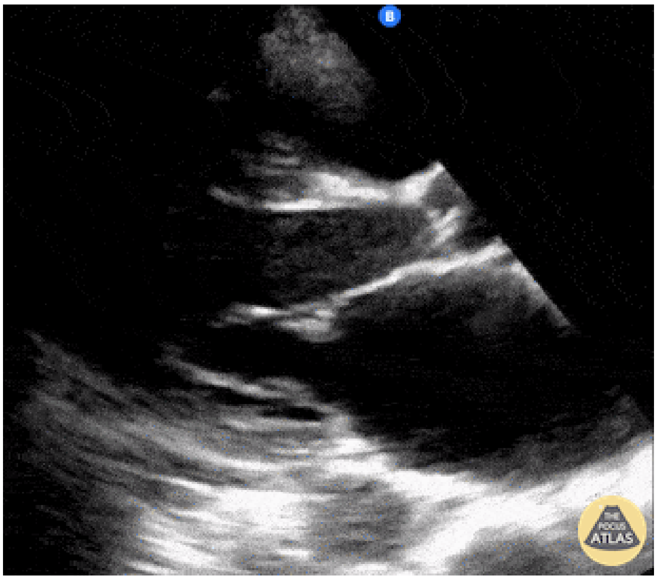

10. Which is shown?

◯ Valvular vegetation
◯ Ascending aortic aneurysm
◯ Left atrial enlargement
◯ Aortic dissection

Please use the following image to answer question number 11.

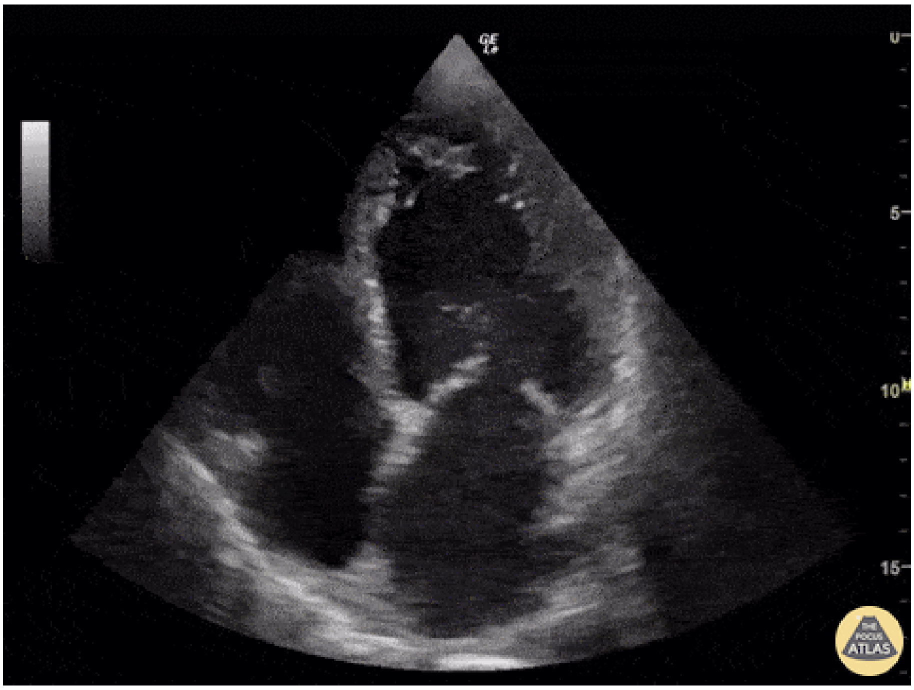

11. What is shown?

- ◯ Intracardiac mass
- ◯ Tricuspid valve vegetation
- ◯ Patent foramen ovale
- ◯ Takotsubo cardiomyopathy

Please use the following image to answer question number 12.

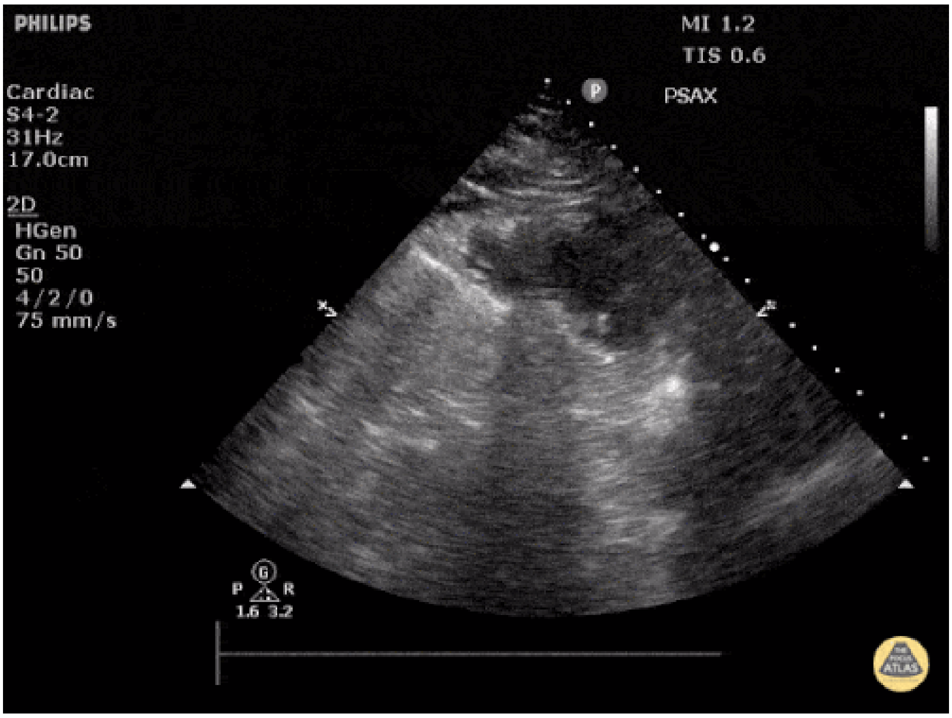

12. Which is demonstrated?

- ◯ Regional wall motion abnormality
- ◯ Cardiac tamponade
- ◯ Hyperdynamic left ventricular function
- ◯ Normal image

Please use the following image to answer question number 13.

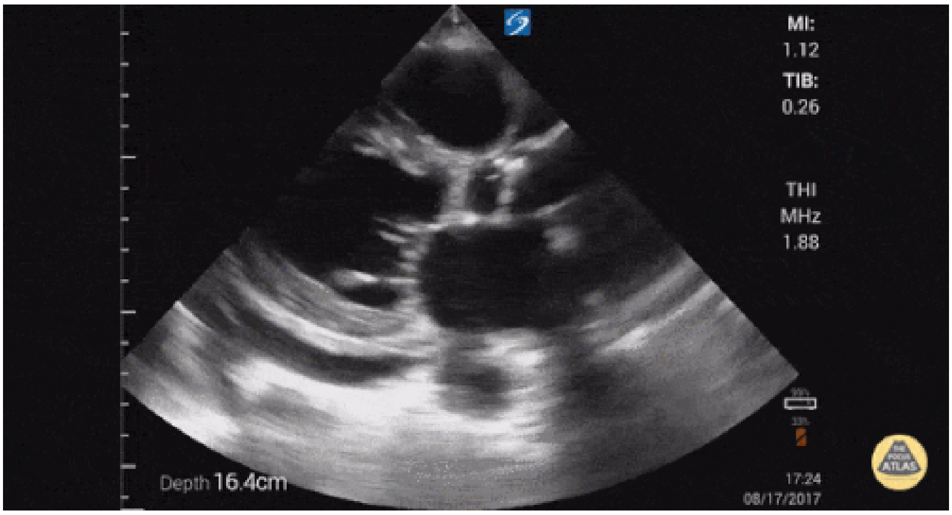

13. Which of the following are seen in the above image?

- ◯ Normal left ventricular systolic function
- ◯ Descending thoracic aorta
- ◯ Cardiac tamponade
- ◯ Pleural effusion

Please use the following image to answer question number 14.

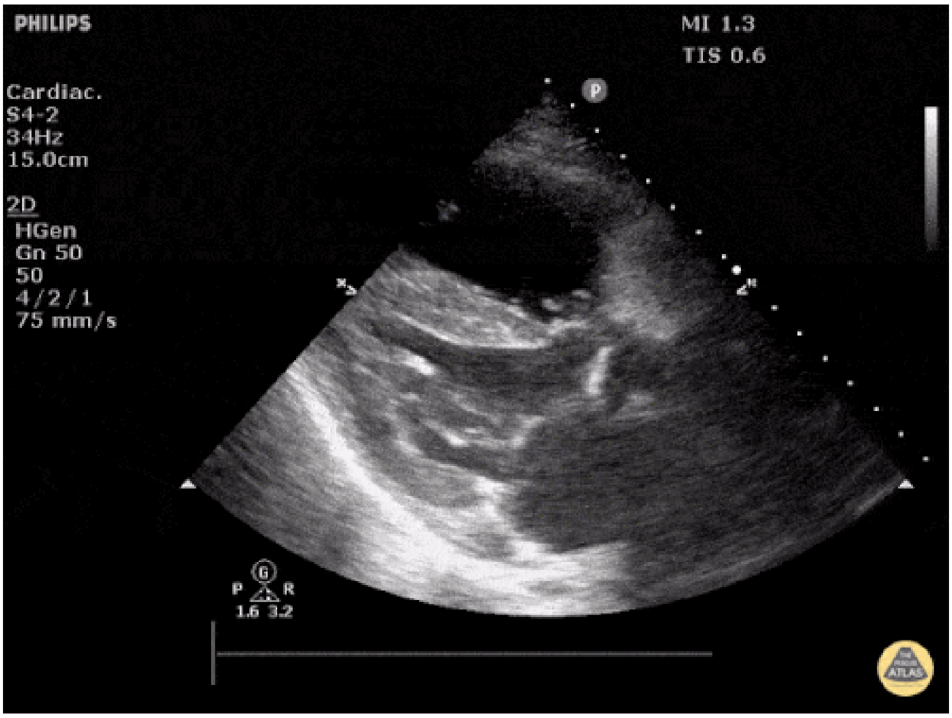

14. Which are suggested by the above image?

- ◯ Endocarditis
- ◯ Right ventricular dysfunction
- ◯ Cardiomyopathy
- ◯ Severe left ventricular hypertrophy

Please use the following image to answer question number 15.

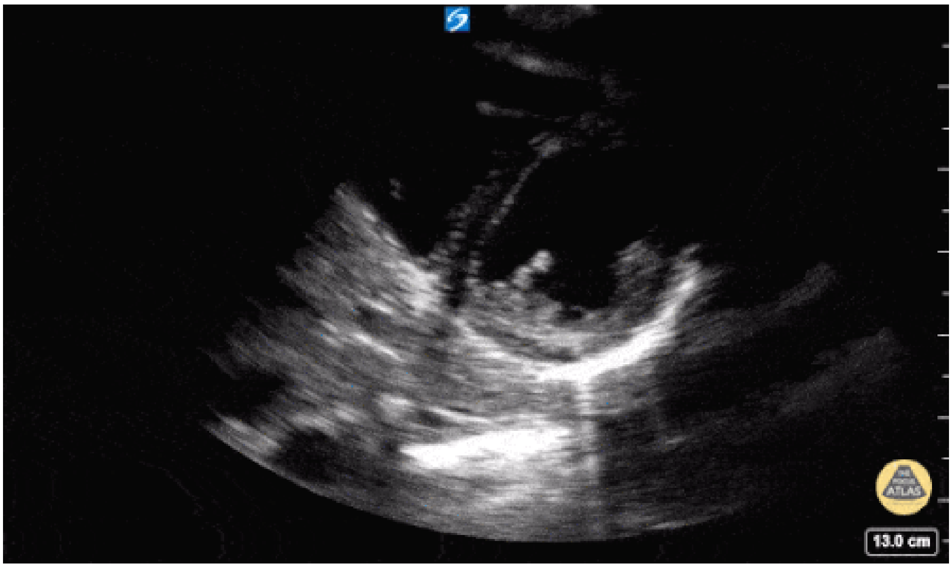

15. Which best describes the left ventricular function?

- ◯ Normal
- ◯ Hyperdynamic
- ◯ Reduced
- ◯ Severely reduced
