# Supplementary material for: A randomized controlled trial of curated X exposure for cardiac point of care ultrasound education
Source: BMC Med Educ. 2025 Dec 5;26:42. doi: 10.1186/s12909-025-07385-3 (PMC12797907; doi:10.1186/s12909-025-07385-3)
Supplement: Supplementary file 2 — Supplementary Material 2 [file 12909_2025_7385_MOESM2_ESM.docx]

# Intervention Arm

1. NephroPOCUS @NephroP
2. TPA @thepocusatlas
3. Society of point-of-care ultrasound (SPOCUS) @POCUS_Society
4. The Echo Lady @The_echo_lady
5. Daniel Opazo #POCUS @TaotePOCUS
6. POCUS Med Ed @pocusmeded
7. Emergency Echo @EmergencyEcho
8. POCUS Bot @POCUSbot
9. Global Ultrasound Institute @globalultrasou1
10. Robert Jones @RJonesSonoEM

# Control Arm

1. The Cochrane Library @CochraneLibrary
2. The BMJ @bmj_latest
3. JAMA @JAMA_current
4. NEJM @NEJM
5. Nature Medicine @NatureMedicine
6. AMA @AmerMedicalAssn
7. NEJM Journal Watch @JWatch
8. The Lancet @TheLancet
9. CDC @CDCgov
10. UpToDate @UpToDate
